# Comment on “Theoretical studies on a carbonaceous molecular bearing: association thermodynamics and dual-mode rolling dynamics” by H. Isobe, K. Nakamura, S. Hitosugi, S. Sato, H. Tokoyama, H. Yamakado, K. Ohno and H. Kono, *Chem. Sci.*, 2015, **6**, 2746†
†Electronic supplementary information (ESI) available: Association free energies using B97-D2/def2-TZVP geometries. Results of different implicit solvation models. Gas phase association energies with LC-BLYP/6-311G* geometries. Geometry of complexes. See DOI: 10.1039/c5sc04676a


**DOI:** 10.1039/c5sc04676a

**Published:** 2016-02-09

**Authors:** Enrique M. Cabaleiro-Lago, Jesús Rodríguez-Otero, Adrià Gil

**Affiliations:** a Departamento de Química Física , Facultad de Ciencias , Universidade de Santiago de Compostela , Campus de Lugo , Av. Alfonso X El Sabio, s/n , 27002 Lugo , Galicia , Spain . Email: caba.lago@usc.es; b CIQUS and Facultade de Química (Departamento de Química Física) , Universidade de Santiago de Compostela , 15782 Santiago de Compostela , Galicia , Spain . Email: r.otero@usc.es; c Centro de Química e Bioquímica , DQB , Faculdade de Ciências , Universidade de Lisboa , Campo Grande , 1749-016 Lisboa , Portugal

## Abstract

The LC-BLYP functional accompanied with proper calculations leads to unreliable results for systems governed by π···π interactions. It seems quite clear that a good representation of dispersion interactions is required, so DFT must be supplemented (through the DFT-D formalism or the many-body dispersion method) in order to afford good results.

## Abstract 2

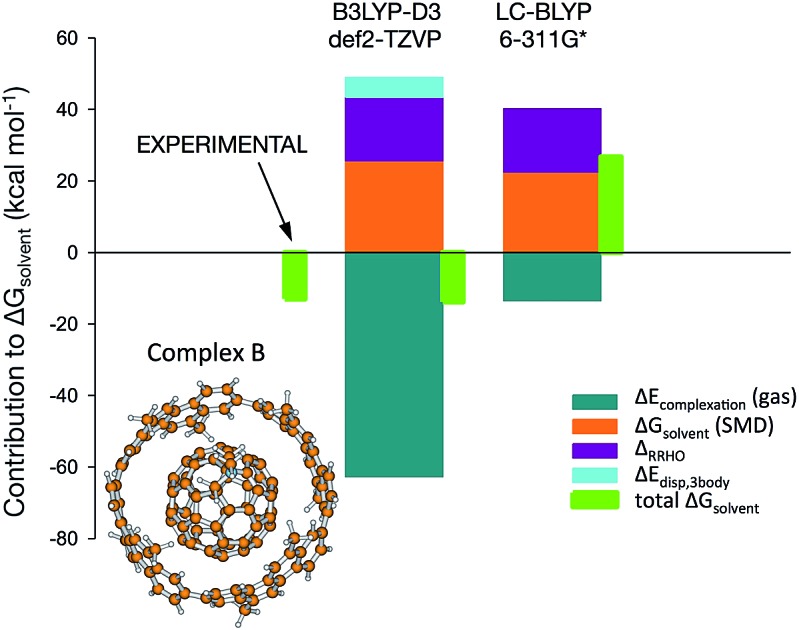
The LC-BLYP functional leads to unreliable results for systems governed by π···π interactions.



## 

In a recent article Isobe *et al.*^1^ have presented a theoretical study on the association thermodynamics of molecular peapods comprising a tubular molecule and a fullerene derivative. As an important part of their study, an evaluation of different functionals is performed, reaching the conclusion that the most appropriate functionals in order to reproduce the experimental association enthalpy of the complexes are BMK and especially LC-BLYP. On the contrary, these authors conclude that dispersion-corrected functionals (DFT-D) provide highly overestimated values, with differences around 50 kcal mol^–1^ to experiment, and that a parameterized functional which accounts for some dispersion (M06-2X) also leads to a substantial overestimation (above 30 kcal mol^–1^). This surprising conclusion (taking into consideration that the interaction in this kind of systems is dispersion-controlled and LC-BLYP does not reproduce dispersion properly) could seem correct at first glance. However, it is not stated throughout the paper that this is a consequence of a huge, and we think rather fortuitous, cancellation of different terms contributing to the final association energies. Furthermore, there is no reason why this behaviour (observed by comparing to a single experimental value) could be extrapolated to other systems. So, concluding that this study could serve as a benchmark for theoretical studies on curved π-systems, and that LC-BLYP is the method of choice, seems too far-fetched to us. Therefore, according to our opinion, the paper is flawed in several crucial aspects of the calculations and the interpretation of their results.

A first criticism to the paper of Isobe *et al.* relates to Table 1.^1^ It lists the results obtained for the association energies of the complex *in vacuo* and in solvent (employing a polarizable continuum model, PCM) with a variety of functionals. The results obtained are thus compared with the experimental association enthalpy (–12.5 kcal mol^–1^) in order to assess the performance of the functionals tested. In our opinion, this procedure is not appropriate, since the magnitudes compared have different nature. Values of (Δ*E* + PCM) include the gas-phase association energy plus the correction to the Gibbs free energy due to the solvent, so there is some mixing between electronic energies and Gibbs free energies. Furthermore, this combination is compared to the experimental enthalpy of association so that an improper deviation is listed in the last column of Table 1 in the original paper. Therefore, even though (Δ*E* + PCM) and Δ*H* could be numerically similar, they should not be compared directly as in Table 1 of ref. 1, and especially this comparison should not be used in order to assess the performance of the different methods. This inadequate comparison is what yields LC-BLYP as the best method and large overestimations using dispersion-corrected functionals, leading the authors to conclude that “the dispersion effects at the curved π-interfaces are overestimated by the present DFT methods with pairwise dispersion forces” and “the results may indicate that further improvements in the theoretical models of dispersion forces are necessary especially for the curved π-systems”. Even though pairwise dispersion models are known to overestimate dispersion contribution in large systems, the error is by far much smaller than the deviations shown in Table 1 of ref. 1 (the three-body corrections amount to around 3–5 kcal mol^–1^ as shown by Grimme in similar systems).^2^,^3^

**Table 1:** Calculated association free energy in dichloromethane, Δ*G*_solvent_. LC-BLYP/6-311G* geometries are used for the complexes. Single point calculations are performed with the def2-TZVP basis set, except for the LC-BLYP functional where the 6-311G* one is employed. All values in kcal mol^–1^

	Complex	Δ*E*_comp_^*a*^	Δ*E*_disp,3body_	*Δ*_RRHO_ ^*b*^	Δ*G*_gas_^*c*^	Δ*G*_solvent(SMD)_	Δ*G*_solvent_	dev^*f*^
B3LYP-D3	A	–60.49 (–80.64)	5.72	18.63	–36.14	23.70	–12.44	
	B	–62.37 (–82.05)	5.45	17.69	–39.23	24.49	–14.74	–1.74
B97-D2	A	–64.75 (–92.81)	5.72^*d*^	18.63	–40.40	23.45	–16.96	
	B	–65.92 (–94.23)	5.45^*d*^	17.69	–42.78	24.16	–18.62	–5.62
TPSS-D3	A	–57.43 (–73.43)	5.72	18.63	–33.08	23.38	–9.70	
	B	–59.09 (–74.47)	5.45	17.69	–35.95	24.08	–11.88	+1.12
B97-D3	A	–64.18 (–92.25)	5.72	18.63	–39.83	23.45	–16.39	
	B	–65.91 (–94.22)	5.45	17.69	–42.77	24.16	–18.61	–5.61
LC-BLYP	A^*e*^	–12.01	—	18.63	6.62	21.42	28.05	
	B	–13.47	—	17.69	4.22	22.47	26.69	+39.69

^*a*^Δ*E*_complexation_ includes Δ*E*_dispersion_ contribution (in brackets).

^*b*^Frequencies obtained at the PM6-D3 level.

^*c*^Δ*G*_gas_ = Δ*E*_comp_ + Δ*E*_disp,3body_ + *Δ*_RRHO_.

^*d*^For B97-D2 a 3-body term equal to that of the other functionals is assumed.

^*e*^Geometry taken from Isobe *et al.* data.^1^

^*f*^Deviation relative to the experimental value of –13.0 kcal mol^–1^ for complex B.^8^

Also, the procedure for obtaining the final values in solution needs clarification. Most often, association thermodynamics in solution is discussed in terms of association Gibbs energies, directly related to the association equilibrium constants because obtaining entropy and enthalpy contributions with similar robustness has proven challenging.^4^,^5^ To this end, the theoretical estimation of Gibbs energies is usually based on the following expression:

1 *G*0solv = *E*_gas_ + *G*_gas,RRHO_ + Δ*G*0solv

where *E*_gas_, *G*_gas,RRHO_ and Δ*G*0solv are the gas-phase electronic energy, the rigid-rotor harmonic oscillator contribution, and the solvation free energy, respectively. The calculation of each of these terms faces problems as recently reviewed by Jensen,^6^ but a proper selection of procedure and calculation level gives values reasonably close to the experimental ones, as shown in the extensive benchmark by Grimme.^3^ In fact, the apparent success of LC-BLYP relies on the cancelation of the contributions coming from the RRHO term, dispersion (both effects not included in ref. 1) and the description of solvent effects.

In order to shed light upon the different contributions to the stability in this kind of complexes, calculations have been carried out to obtain the different contributions to the association. Since no experimental value for the Gibbs energy is available for the (*P*)-(12,8)-[4]-cyclo-2,8-crysenylene ([4]CC) and fullerenopyrrolidine complex of ref. 1 (complex A, Fig. 1), a virtually identical complex employing unsubstituted fullerene has been also studied (complex B, Fig. 1).^7^ For this latter complex, Isobe *et al.* determined in a recent previous work an association Gibbs energy of –13.0 ± 0.3 kcal mol^–1^.^8^

**Fig. 1:**
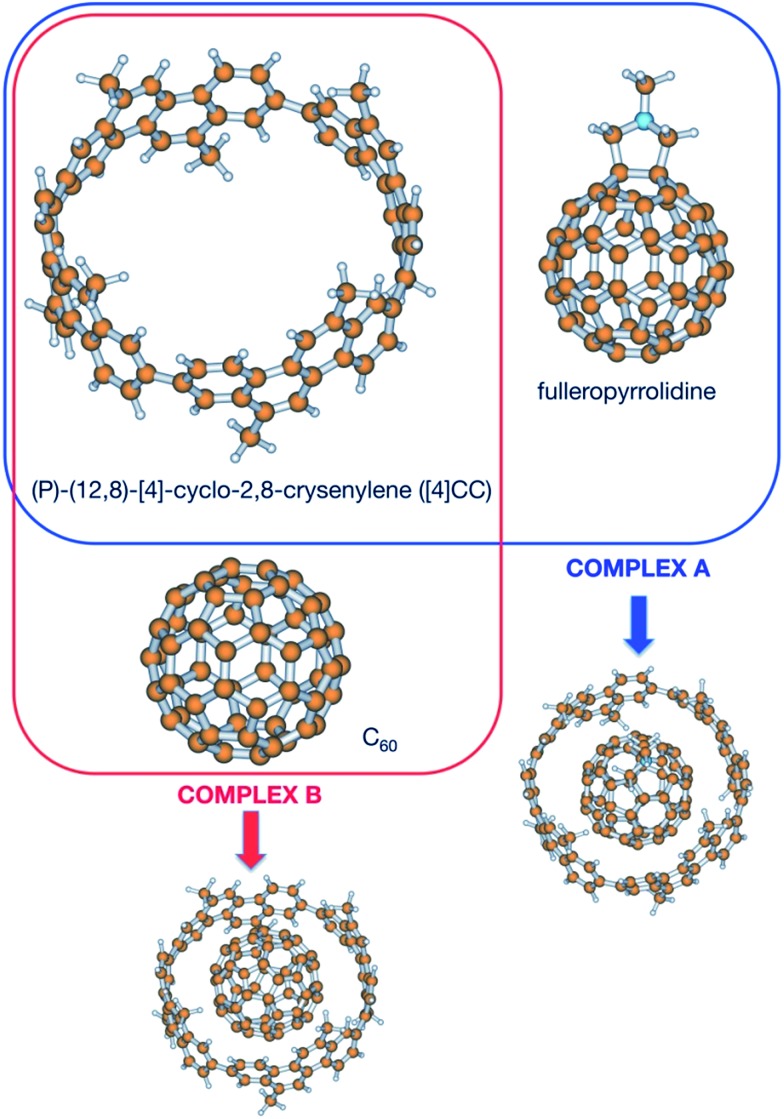
Species considered in this work.

Table 1 shows the different contributions to the association Gibbs energy for both complexes employing the LC-BLYP/6-311G* optimized structures (values obtained with the B97-D2/def2-TZVP geometries are listed in Table S1,† showing a similar behaviour). Considering the gas phase results, the stabilization of the complexes mostly comes from dispersion, as expected for this kind of complexes based on π···π interactions. The 3-body contribution to dispersion amounts to around 5–6 kcal mol^–1^, in agreement with Grimmes's results^2^,^3^ (to some extent, this contribution takes care of the overestimation introduced by purely pairwise additive models). As a consequence of the great similarity of the two systems considered, the association energies in the gas phase differ only by 2 kcal mol^–1^ at most, always favouring the complex with pristine C_60_ (complex B). DFT-D calculations produce association energies in the gas phase much larger than the experimental Gibbs energies, as expected. Therefore, the contributions from solvent and RRHO in eqn (1) must destabilize the complex in order to obtain values closer to experiment.

In ref. 1, solvent effects modelled with the IEFPCM formalism amount to 2.8 kcal mol^–1^ and are added to the gas phase results in order to compare the values obtained with the experiment. However, the results obtained with the SMD model indicate a much larger effect, around 24 kcal mol^–1^. This discrepancy has its origin in the treatment of non-electrostatic terms, which amount to around 19 kcal mol^–1^ with SMD. If these terms (cavitation, repulsion and dispersion) are included in the IEFPCM calculations, solvent effects are now similar to those obtained with SMD (see Table S2†). Therefore, non-electrostatic terms become a crucial contribution to the effect of the solvent and cannot be ignored. If these terms are included, the called-for agreement of LC-BLYP with experiment holds no more.

As for the RRHO contribution, its calculation is quite costly and poses some problems related to low-frequency modes. However, Grimme has shown that semiempirical methods can provide with reasonable values for this correction.^2^ Therefore, the RRHO term has been estimated at the PM6-D3 level of calculation and amounts to around 18–19 kcal mol^–1^. Therefore, at this point, the results from ref. 1, lacking contributions from RRHO and non-electrostatic solvent effects, do not include destabilizing terms contributing more than 40 kcal mol^–1^. Properly including these corrections in the LC-BLYP results would lead to Δ*G* values close to +30 kcal mol^–1^ suggesting that the complex is not formed at all. It is the dispersion contribution the one to compensate these destabilizing effects thus leading to negative Δ*G*. Although there is some inaccuracy with the RRHO approximation, especially in these systems with very low frequencies and long-range motions, the anharmonicity effects on the Gibbs free energy would probably amount to only a few kcal mol^–1^ (for instance, these effects range from 1 to 3 kcal mol^–1^ in a recent work),^9^ so it would not significantly alter the conclusions achieved.

It can be observed in Tables 1 and S1† that for the complex B, the calculated DFT-D values of Δ*G* in solution (dichloromethane) are reasonably close to the experimental value of –13 ± 0.3 kcal mol^–1^.^8^ The different values obtained come from the differences observed for the association energies in the gas phase. The behaviour observed for the complex of ref. 1 (complex A) is similar, with predicted values for Δ*G* around 2 kcal mol^–1^ smaller than those of complex B, and compatible with the experimental value of –12.5 kcal mol^–1^ obtained for Δ*H*.^1^ The calculations predict therefore complex formation in both cases, being the complexation of C_60_ somewhat more favoured. On the other hand, LC-BLYP predicts values for Δ*G* in solution around +30 kcal mol^–1^ and no complex formation. Considering that LC-BLYP greatly fails describing the values for Δ*G* it must be considered that any coincidence with the experimental Δ*H* is totally fortuitous and therefore, LC-BLYP should not be employed for studying interactions involving curved π structures.

As commented above, the purely pairwise models tend to overestimate dispersion effects in large systems. For that reason, we have also tested an alternative method that includes many body effects, namely the Many Body Dispersion (MBD) model.^10^–^12^ This method includes terms beyond the standard three-body Axilrod–Teller contribution to dispersion, and has shown a remarkable performance for obtaining accurate gas-phase complexation energies in large complexes.^13^–^15^ Indeed, the calculations with this approach lead to a slightly less negative association energy than that obtained with DFT-D methods of Table 1 (see Table S3†). However, the PBE + MBD association energy is more negative (around 3 kcal mol^–1^) than that obtained with PBE-D3, which suggest that the addition of just the three-body contribution leads to an underestimated dispersion. Considering the MBD-corrected gas phase association energy of Table S3,† taking into account the RRHO effects and an average value of Δ*G*_solvent(SMD)_ (23 and 24 kcal mol^–1^ for complex A and B, respectively), the final Δ*G*_solvent_ are –8.89 and –9.59 kcal mol^–1^, for complex A and B, respectively, with a deviation of +3.41 kcal mol^–1^ with respect to the experimental value (–13.0 kcal mol^–1^) obtained for complex B. That is to say, DFT-D and DFT + MBD predictions are fairly close (within a few kcal mol^–1^) and all of them very far from those of LC-BLYP (several tens of kcal mol^–1^).

Regarding the performance of the different DFT-D methods, it is clear that differences among them are logically triggered by the complexation energy in gas phase (the same RRHO is employed whereas Δ*G*_solvent(SMD)_ is almost identical for different functionals). Taking mainly into account the data of Table S1† (which corresponds to a DFT-D geometry), the best result corresponds to B3LYP-D3, whereas B97-D2 and especially B97-D3 lead to a slight overestimation of the stability of complexes. This fully agrees with previous calculations for the corannulene dimer,^16^ the typical complex governed by π···π interactions between curved systems. Under this circumstances using B97-D2 could be a good choice considering that pretty reasonable results are obtained, with huge savings of computation time regarding to hybrid functionals like B3LYP-D3 if the resolution of identity approximation (RI^17^) is applied.^18^–^23^ Meanwhile, TPSS-D3 leads to a slight underestimation of the association free energy of the complex. However, all these conclusions should be taken with much caution because several contributions to the total Δ*G*_solvent_ have not been rigorously calculated but are included only as reasonable estimations (like *Δ*_RRHO_ or Δ*G*_solvent(SMD)_).

Therefore, as commented above and as suggested by Grimme among others, the description of vdW interactions needs a good representation of dispersion interactions, so DFT must be supplemented in order to provide good dispersion estimations.^24^ Only a proper description of dispersion, plus appropriate treatment of RRHO effects and solvent could lead to a good description of the system. Relying on a fortuitous agreement of a method that does not properly include the correct physics of the problem could lead to huge errors in other systems.

Finally, it is noteworthy that although the theoretical reproduction of the experimental association free energy has been greatly improved in recent times,^3^,^25^ it has not yet achieved a level of quality equivalent to that obtained for gas phase calculations. However, the existence of benchmarks in gas phase (at the CCSD(T)/QCISD(T) level, see Table 2) allows to check accurately the behaviour of different functionals. Not surprisingly, LC-BLYP performs very poorly when applied to some simple examples of systems governed by π···π dispersion. This happens both for dimers between planar monomers and for the dimer between curved monomers (corannulene dimer, to our knowledge, the only case of this type with an accurate reference value). Oppositely, a dispersion-corrected functional (specifically B97-D2, but it could be another one, as BLYP-D2, BP86-D2, …) gives rise to rather acceptable results (maximum deviation of about 10%). In the light of the results of Table 2, it is noteworthy that LC-BLYP gives rise to exceptionally bad results when the interaction takes place between structures with eclipsed bonds (naphthalene, coronene, and corannulene dimers).

**Table 2:** Gas phase complexation energy for several complexes with π···π interaction. All values in kcal mol^–1^

	QCISD(T)/CCSD(T)	LC-BLYP/6-311G*	B97-D2/TZVP^*f*^
Benzene dimer (*C*_2h_)	–2.62^*a*^, –2.65^*b*^	–0.37	–2.46
Pyrazine dimer (*C*_s_)	–4.20^*a*^, –4.26^*b*^	–1.78	–3.81
Adenine···thymine stack	–11.66^*a*^, –11.86^*b*^	–5.75	–11.33
Naphthalene dimer (*D*_2h_)	–3.78^*c*^	0.64	–4.12
Coronene dimer (*D*_6h_)	–14.73^*d*^	–0.27	–16.30
Corannulene dimer (*C*_5v_)	–15.50^*e*^	–2.36	–16.06

^*a*^ Ref. 26.

^*b*^ Ref. 27.

^*c*^ Ref. 28.

^*d*^ Ref. 29.

^*e*^ Ref. 30.

^*f*^ Ref. 23.

In summary, according to our results, the apparent good performance of the LC-BLYP/6-311G** method obtained by Isobe *et al.*^1^ for reproducing the experimental association enthalpy of complex A is just a fortuitous result of a huge cancellation of different large contributions. Two factors must be clearly stressed: first, the lack of thermodynamic corrections through harmonic frequencies and second, the poor description of the effect of the solvent. This leads to a simple question: is there any certainty for this cancellation to take place in other π···π complexes? We believe that expecting such large error compensations without considering the physics behind the problem would be “walking on incredibly thin ice”.

## Computational details

The structure of the complex between (*P*)-(12,8)-[4]-cyclo-2,8-crysenylene and fulleropyrrolidine (complex A) has been taken from ref. 1. Complex B has been constructed from complex A just deleting the extra atoms from the fulleropyrrolidine. The structures of the isolated molecules and their complexes have been optimized at the LC-BLYP/6-311G* and B97-D2/def2-TZVP levels of calculation.

Counterpoise-corrected complexation energies are then obtained with Gaussian 09 (ref. 31) at the LC-BLYP/6-311G* level for consistency with the original paper. Complexation energies are also obtained with different dispersion-corrected functionals: B97-D2 (using the old dispersion correction from Grimme),^32^,^33^ TPSS-D3, and B3LYP-D3 (with the D3 correction and Becke–Johnson damping function)^34^,^35^ using Orca 3.0.3.^36^ Solvent effects have been estimated for each of these functionals by using the universal solvation model SMD^37^ based on COSMO^38^ charges as implemented in Orca (for LC-BLYP the corresponding calculation is performed using Gaussian 09). The resolution of the identity approximation using the def2-TZVP/J auxiliary basis set has been employed in all calculations done with Orca. The RI approach is employed in B97-D2 and TPSS-D3 calculations, whereas the RIJCOSX approach has been employed in order to save time in B3LYP-D3 calculations.^39^

The rigid rotor harmonic oscillator correction to the energies is obtained from frequencies at the PM6-D3 semiempirical level with MOPAC2012.^40^

MBD calculations has been performed at the PBE/TZP level using the ADF program.^41^

## Supplementary Material

Supplementary information
