# Supplementary material for: Comment on “Theoretical studies on a carbonaceous molecular bearing: association thermodynamics and dual-mode rolling dynamics” by H. Isobe, K. Nakamura, S. Hitosugi, S. Sato, H. Tokoyama, H. Yamakado, K. Ohno and H. Kono, Chem. Sci., 2015, 6, 2746
Source: Chem Sci. 2016 Feb 9;7(4):2924–8. doi: 10.1039/c5sc04676a (PMC6054031; doi:10.1039/c5sc04676a)
Supplement: Supplementary file 1 [file SC-007-C5SC04676A-s001.pdf]

Electronic Supplementary Material (ESI) for Chemical Science.
This journal is © The Royal Society of Chemistry 2016

Comment on "Theoretical studies on a carbonaceous molecular bearing: association thermodynamics and dualmode rolling dynamics"

Enrique M. Cabaleiro-Lago, Jesús Rodríguez-Otero and Adrià Gil

Supplementary Information

Table S1 2
Table S2 2
Table S3 3
Geometry of complexes 4

Table S1. Calculated association free energy in dichloromethane, $\Delta G_{solvent}$. B97-D2/def2-TZVP geometries are used for the complexes. Single point calculations are performed with the def2-TZVP basis set. All values in kcal mol^{-1}.

	complex	ΔE_{comp} [a]	$\Delta E_{disp,3body}$	Δ_{RRHO} [b]	ΔG_{gas} [c]	$\Delta G_{solv.\ (SMD)}$	$\Delta G_{solv.}$	dev[e]
B3LYP-D3	A	-62.10 (-86.80)	5.85	18.63	-37.62	25.40	-12.22	
	B	-62.72 (-86.28)	5.56	17.69	-39.47	25.65	-13.83	-0.83
B97-D2	A	-62.95 (-98.28)	5.85[d]	18.63	-38.47	25.10	-13.37	
	B	-63.37 (-97.42)	5.56[d]	17.69	-40.12	25.28	-14.83	-1.83
TPSS-D3	A	-58.22 (-78.53)	5.85	18.63	-33.74	25.06	-8.68	
	B	-58.88 (-77.99)	5.56	17.69	-35.63	25.24	-10.39	+2.61
B97-D3	A	-64.95 (-100.28)	5.85	18.63	-40.47	25.10	-15.36	
	B	-65.68 (-99.73)	5.56	17.69	-42.43	25.28	-17.14	-4.14

[a] $\Delta E_{complexation}$ includes $\Delta E_{dispersion}$ contribution (in brackets).
[b] Frequencies obtained at the PM6-D3 level.
[c] $\Delta G_{gas} = \Delta E_{comp} + \Delta E_{disp,3body} + \Delta_{RRHO}$.
[d] For B97-D2 a 3-body term equal to that of the other functionals is assumed.
[e] Deviation relative to the experimental value of -13.0 kcal mol^{-1} for complex B.[8]

Table S2. $\Delta G_{solvent}$ calculated with different implicit solvation models (in kcal mol^{-1}). LC-BLYP/6-311G* geometry is used, and to make a fair comparison, all single point calculations are performed with the same basis set, namely, the LC-BLYP/6-311G*.

	electrostatic	non-electrostatic terms[a]	total	
SMD	2.73	18.69	21.42	complex A
PCM (IEFPCM)	2.72[b]	20.01	22.73	
CPCM/COSMO	3.35	20.01	23.36	
SMD	3.17	19.30	22.47	complex B
PCM (IEFPCM)	3.01	16.70	19.70	
CPCM/COSMO	3.90	16.70	20.60	

[a] Dispersion, repulsion and cavitation energies. The SMD model includes implicitly the non-electrostatic terms of Truhlar and co-workers. For PCM and CPCM models, the non-electrostatic terms must be explicitly added through the READ option (keywords Dis, Rep, and Cav). Curiously, in the previous version of Gaussian (G03), the non-electrostatic terms are calculated as default.
[b] 2.8 kcal mol^{-1} is is the value supplied by Isobe *et al.* as the PCM solvent effect.

Table S3. Gas phase association energy (in kcal mol[-1]). For DFT-D models it corresponds to ΔE_{comp} + $\Delta E_{disp,3body}$, taken from Table 1 of the manuscript. LC-BLYP/6-311G* geometries are used.

B3LYP-D3	A<br>B	-54.77<br>-56.92
B97-D2	A<br>B	-59.03<br>-60.47
TPSS-D3	A<br>B	-51.71<br>-53.64
B97-D3	A<br>B	-58.46<br>-60.46
PBE-D3[a]	A<br>B	-47.54<br>-48.86
PBE+MBD[b]	A<br>B	-50.52<br>-51.28
LC-BLYP	A<br>B	-12.01<br>-13.47

[a] Calculation with the TZP basis set. The D3 dispersion is -56.65 and -57.31 kcal mol[-1], for complex A and B, respectively. After adding $\Delta E_{disp,3body}$, these dispersions become -50.93 and -51.86 kcal mol[-1], respectively.
[b] Calculation with the TZP basis set. The MBD dispersion is -53.91 and -54.28 kcal mol[-1], for complex A and B, respectively.

According to the above values of dispersion, and taking the Many Body Dispersion values as reference, it seems clear that the pairwise models from Grimme tend to overestimate dispersion effects in large systems. The addition of the three-body Axilrod-Teller contribution removes the overestimation but leads to an overcorrection, so that at the end the dispersion is underestimated. Therefore, this points to the need to go beyond the three-body contributions, including four-body contributions and more. In fact, the series only starts to converge with six-body contributions.[14]

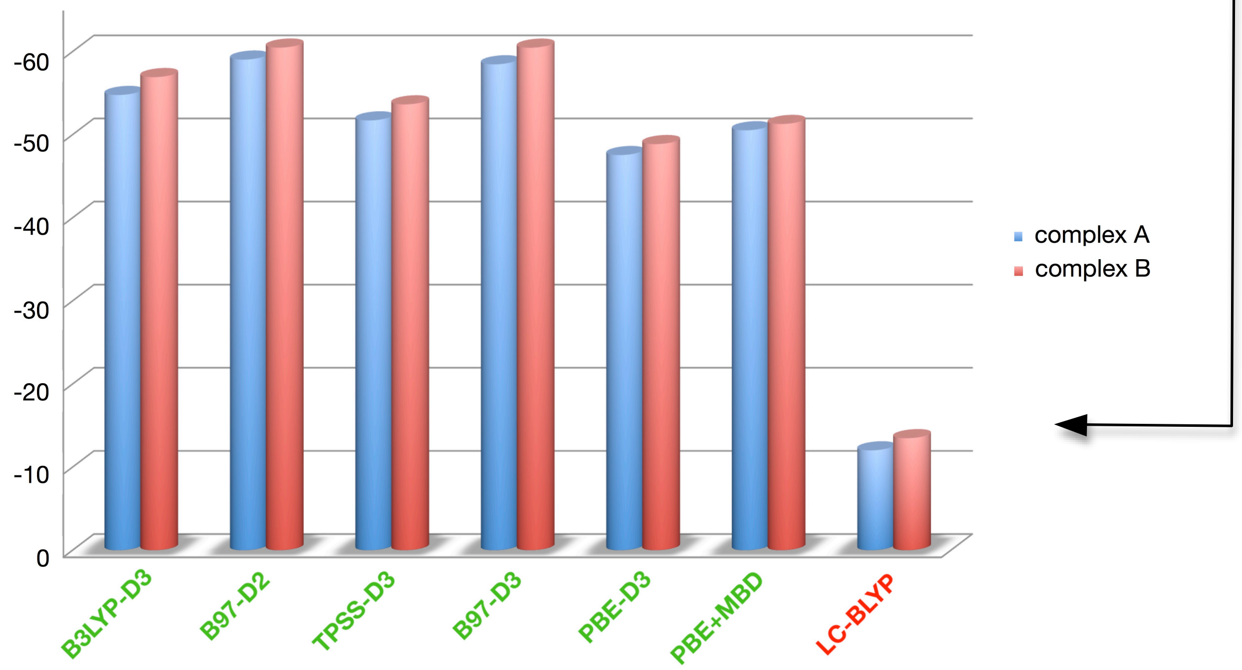

Complex B (LC-BLYP/6-311G*)

C	5.541847	4.158322	-0.179813
C	4.861953	4.647059	-1.259396
C	3.788850	5.542386	-1.131608
C	3.370300	5.926798	0.143782
C	4.207236	5.585742	1.223135
C	5.258806	4.741486	1.068730
C	3.025149	5.943182	-2.277085
C	2.050161	6.476340	0.316873
C	1.253706	6.681926	-0.793015
C	1.820581	6.507426	-2.087715
C	-0.164842	6.852319	-0.619955
C	-0.718768	6.727046	0.654960
C	0.143740	6.715142	1.799660
C	1.471115	6.613964	1.610281
H	5.054921	4.236289	-2.240277
H	3.989327	5.958384	2.215063
H	5.850513	4.477040	1.936568
H	1.234591	6.764797	-2.960298
H	2.107943	6.555387	2.483421
C	-2.089056	6.443212	0.780603
H	-2.455619	6.173417	1.761131
C	-1.060356	6.952007	-1.702075
H	-0.689777	7.179474	-2.692523
C	-2.911288	6.319719	-0.302553
C	-2.384907	6.704385	-1.550444
H	-3.031103	6.752836	-2.418299
C	-4.175946	5.555501	-0.199096
C	-4.651020	4.870921	-1.281369
C	-4.774649	5.279212	1.043241
C	-5.546447	3.796817	-1.159339
H	-4.226566	5.059100	-2.257594
C	-5.619293	4.227269	1.192494
H	-4.520664	5.875631	1.911046
C	-5.946269	3.384373	0.113298
C	-5.928907	3.025058	-2.305538
H	-6.003837	4.013778	2.180833
C	-6.498874	2.065985	0.286907
C	-6.491866	1.819741	-2.116095
C	-6.684574	1.261865	-0.820530
C	-6.661792	1.496351	1.581759
H	-6.734486	1.226912	-2.988334
C	-6.849904	-0.156142	-0.640004
C	-6.756670	0.170169	1.779263
H	-6.625966	2.140354	2.450885
C	-6.739377	-0.700680	0.640677
C	-6.934290	-1.059510	-1.716348
C	-6.451604	-2.068970	0.779290
H	-7.150681	-0.696712	-2.712148
C	-6.686301	-2.382646	-1.551843
H	-6.194048	-2.427950	1.765998
C	-6.313245	-2.898925	-0.296765
H	-6.723291	-3.034281	-2.416007
C	6.313245	2.898925	-0.296765
C	6.451604	2.068970	0.779290
C	6.686301	2.382646	-1.551843
C	6.739377	0.700680	0.640677
H	6.194048	2.427950	1.765998
C	6.934290	1.059510	-1.716348
H	6.723291	3.034281	-2.416007
C	6.849904	0.156142	-0.640004
C	6.756670	-0.170169	1.779263
H	7.150681	0.696712	-2.712148
C	6.684574	-1.261865	-0.820530
C	6.661792	-1.496351	1.581759
C	6.498874	-2.065985	0.286907
C	6.491866	-1.819741	-2.116095
H	6.625966	-2.140354	2.450885
C	5.946269	-3.384373	0.113298
C	5.928907	-3.025058	-2.305538
H	6.734486	-1.226912	-2.988334
C	5.546447	-3.796817	-1.159339
C	5.619293	-4.227269	1.192494
C	4.651020	-4.870921	-1.281369
H	6.003837	-4.013778	2.180833
C	4.774649	-5.279212	1.043241
H	4.226566	-5.059100	-2.257594
C	4.175946	-5.555501	-0.199096
H	4.520664	-5.875631	1.911046
C	-5.541847	-4.158322	-0.179813
C	-4.861953	-4.647059	-1.259396
C	-5.258806	-4.741486	1.068730
C	-3.788850	-5.542386	-1.131608
H	-5.054921	-4.236289	-2.240277
C	-4.207236	-5.585742	1.223135
H	-5.850513	-4.477040	1.936568
C	-3.370300	-5.926798	0.143782
C	-3.025149	-5.943182	-2.277085
H	-3.989327	-5.958384	2.215063
C	-2.050161	-6.476340	0.316873
C	-1.820581	-6.507426	-2.087715
C	-1.253706	-6.681926	-0.793015
C	-1.471115	-6.613964	1.610281
H	-1.234591	-6.764797	-2.960298
C	0.164842	-6.852319	-0.619955
C	-0.143740	-6.715142	1.799660
H	-2.107943	-6.555387	2.483421
C	0.718768	-6.727046	0.654960
C	1.060356	-6.952007	-1.702075
C	2.089056	-6.443212	0.780603
H	0.689777	-7.179474	-2.692523
C	2.384907	-6.704385	-1.550444
H	2.455619	-6.173417	1.761131
C	2.911288	-6.319719	-0.302553
H	3.031103	-6.752836	-2.418299
C	5.641623	-3.530222	-3.682693
H	4.569472	-3.533247	-3.892822
H	6.003153	-4.550132	-3.822947
H	6.117903	-2.900661	-4.432643
C	3.537108	5.671214	-3.654568
H	3.537226	4.601329	-3.875953
H	4.559060	6.030647	-3.784991
H	2.913331	6.158365	-4.402367
C	-0.435094	6.729423	3.177089
H	-0.943267	5.788630	3.403439

H	-1.163669	7.531825	3.302756
H	0.345403	6.865841	3.923990
C	-5.641623	3.530222	-3.682693
H	-4.569472	3.533247	-3.892822
H	-6.003153	4.550132	-3.822947
H	-6.117903	2.900661	-4.432643
C	-6.794945	-0.399599	3.160170
H	-5.855796	-0.899818	3.409782
H	-7.594845	-1.132795	3.274576
H	-6.951934	0.385042	3.898659
C	-3.537108	-5.671214	-3.654568
H	-3.537226	-4.601329	-3.875953
H	-4.559060	-6.030647	-3.784991
H	-2.913331	-6.158365	-4.402367
C	0.435094	-6.729423	3.177089
H	0.943267	-5.788630	3.403439
H	1.163669	-7.531825	3.302756
H	-0.345403	-6.865841	3.923990
C	6.794945	0.399599	3.160170
H	5.855796	0.899818	3.409782
H	7.594845	1.132795	3.274576
H	6.951934	-0.385042	3.898659
C	-2.102498	1.126804	-2.198566
C	-1.069579	2.132403	-2.198954
C	0.193922	1.815998	-2.622096
C	-1.820903	-0.144781	-2.622237
C	-2.924515	1.332890	-1.032748
C	-2.400464	2.465655	-0.312698
C	-1.253706	2.960220	-1.033009
C	-0.164145	3.427412	-0.349660
C	1.341147	2.311038	-1.903248
C	1.820903	0.144781	-2.622237
C	2.102498	-1.126804	-2.198566
C	1.069579	-2.132403	-2.198954
C	-0.193922	-1.815998	-2.622096
C	-1.341147	-2.311038	-1.903248
C	-2.347460	-1.277840	-1.903266
C	-3.127576	-1.081483	-0.795446
C	-3.423261	0.256770	-0.349638
C	-2.401248	2.466388	1.055302
C	-2.923028	1.332521	1.775083
C	-3.420345	0.256787	1.091073
C	-3.124716	-1.080921	1.536876
C	-2.941471	-1.908269	0.370237
C	-1.986621	-2.886711	0.370429
C	-1.164872	-3.093878	-0.794766
C	0.164145	-3.427412	-0.349660
C	1.253706	-2.960220	-1.033009
C	2.347460	1.277840	-1.903266
C	2.923028	-1.332521	1.775083
C	3.420345	-0.256787	1.091073
C	3.124716	1.080921	1.536876
C	2.346426	1.277244	2.646151
C	1.820580	0.144872	3.366191
C	1.069069	-2.131258	2.941877
C	1.253212	-2.958796	1.775729
C	2.401248	-2.466388	1.055302
C	2.400464	-2.465655	-0.312698
C	2.924515	-1.332890	-1.032748
C	3.423261	-0.256770	-0.349638
C	2.941471	1.908269	0.370237
C	1.986621	2.886711	0.370429
C	1.164751	3.091799	1.536676
C	1.340541	2.310648	2.646227
C	0.194078	1.815711	3.366172
C	0.490789	0.477549	3.811107
C	-0.490789	-0.477549	3.811107
C	-0.194078	-1.815711	3.366172
C	0.163826	-3.424435	1.091409
C	-1.164751	-3.091799	1.536676
C	-1.340541	-2.310648	2.646227
C	-2.346426	-1.277244	2.646151
C	-1.820580	-0.144872	3.366191
C	-2.101494	1.126181	2.940890
C	-1.069069	2.131258	2.941877
C	-1.253212	2.958796	1.775729
C	-0.163826	3.424435	1.091409
C	3.127576	1.081483	-0.795446
C	1.164872	3.093878	-0.794766
C	2.101494	-1.126181	2.940890
C	-0.490718	-0.477507	-3.066286
C	0.490718	0.477507	-3.066286

Complex A (B97-D2/def2-TZVP)

C	-6.963977	1.021723	-0.064362
C	-6.561926	1.789591	1.024994
C	-6.011319	3.080758	0.877555
C	-5.833176	3.610000	-0.439206
C	-6.459443	2.915191	-1.507386
C	-7.014700	1.667872	-1.328872
C	-5.487558	3.798508	2.009453
C	-4.908011	4.699165	-0.642926
C	-4.291290	5.303115	0.482675
C	-4.688311	4.889365	1.790534
C	-3.143249	6.154828	0.283878
C	-2.574562	6.266370	-1.023683
C	-3.299736	5.765432	-2.160203
C	-4.443142	5.039966	-1.948480
H	-6.528316	1.336051	2.009167
H	-6.463558	3.342810	-2.505041
H	-7.438930	1.149323	-2.184708
H	-4.260252	5.382066	2.658332
H	-4.939042	4.619964	-2.818171
C	-1.240119	6.708978	-1.150970
H	-0.766497	6.613567	-2.121475
C	-2.429740	6.754708	1.355103
H	-2.881144	6.817898	2.340416
C	-0.467435	7.064640	-0.049742
C	-1.136897	7.201140	1.196576
H	-0.603259	7.602845	2.054269
C	1.003573	6.994858	-0.140878
C	1.740427	6.592078	0.969273
C	1.680745	7.039926	-1.388586
C	3.034049	6.038086	0.856894
H	1.258726	6.561935	1.940312

C	2.931972	6.482839	-1.533098
H	1.182482	7.459817	-2.258603
C	3.599478	5.860506	-0.445221
C	3.718319	5.511696	2.008255
H	3.384371	6.483084	-2.519685
C	4.698813	4.941611	-0.618869
C	4.812645	4.709707	1.818722
C	5.264489	4.317470	0.522403
C	5.093996	4.493450	-1.915535
H	5.278233	4.276962	2.699119
C	6.119564	3.168714	0.343673
C	5.823618	3.350457	-2.111252
H	4.714498	5.003173	-2.795784
C	6.274168	2.609662	-0.963466
C	6.687592	2.448606	1.427538
C	6.718741	1.275373	-1.085702
H	6.719872	2.893141	2.417457
C	7.145096	1.159034	1.273010
H	6.653545	0.810404	-2.062824
C	7.044945	0.495570	0.020096
H	7.525343	0.623850	2.139294
C	-7.033954	-0.446991	0.064629
C	-6.714080	-1.251725	-1.025158
C	-7.131639	-1.082303	1.332158
C	-6.271289	-2.583800	-0.874660
H	-6.653209	-0.808490	-2.012678
C	-6.674995	-2.368580	1.513831
H	-7.509245	-0.527602	2.187266
C	-6.110896	-3.112539	0.444271
C	-5.828182	-3.352726	-2.006834
H	-6.705216	-2.791806	2.513145
C	-5.252310	-4.254848	0.645748
C	-5.099299	-4.492087	-1.788026
C	-4.695080	-4.907948	-0.483315
C	-4.785149	-4.607071	1.947962
H	-4.726045	-5.023654	-2.657967
C	-3.592682	-5.820234	-0.294492
C	-3.684806	-5.397896	2.149082
H	-5.240810	-4.147763	2.820036
C	-3.013532	-5.958675	1.006398
C	-2.934932	-6.472056	-1.371110
C	-1.718276	-6.508195	1.121416
H	-3.397710	-6.500987	-2.352495
C	-1.681190	-7.022653	-1.223625
H	-1.226036	-6.448667	2.085795
C	-0.992579	-6.941780	0.015800
H	-1.189651	-7.465300	-2.086220
C	6.978172	-0.975738	-0.077646
C	6.583180	-1.722330	1.028949
C	7.023549	-1.647248	-1.329192
C	6.032711	-3.016557	0.909796
H	6.555305	-1.249779	2.004377
C	6.467461	-2.897727	-1.480293
H	7.443902	-1.145976	-2.197135
C	5.846414	-3.570957	-0.395439
C	5.516435	-3.712953	2.058294
H	6.466817	-3.345297	-2.469175
C	4.918992	-4.662746	-0.572931

C	4.715452	-4.807379	1.865041
C	4.309223	-5.244823	0.567736
C	4.443253	-5.025114	-1.868839
H	4.293288	-5.283750	2.744749
C	3.158034	-6.097541	0.392697
C	3.295518	-5.749932	-2.058886
H	4.932798	-4.620883	-2.749531
C	2.578332	-6.229594	-0.908195
C	2.452439	-6.677211	1.479961
C	1.242142	-6.671858	-1.017111
H	2.911723	-6.724183	2.462500
C	1.157679	-7.124114	1.339623
H	0.759841	-6.592999	-1.984926
C	0.478885	-7.008617	0.096490
H	0.629671	-7.509458	2.208247
C	-3.132856	-5.595630	3.538300
H	-2.166804	-5.081548	3.651872
H	-2.961772	-6.658285	3.757652
H	-3.821254	-5.189631	4.287589
C	-5.737496	3.306933	3.412801
H	-5.239773	2.340449	3.583499
H	-6.808836	3.155100	3.602234
H	-5.349900	4.022001	4.146729
C	-2.755159	5.936254	-3.554878
H	-1.834079	5.348440	-3.684805
H	-2.503340	6.984348	-3.765393
H	-3.482756	5.593420	-4.298471
C	3.188629	5.762387	3.397583
H	2.216210	5.268302	3.542087
H	3.035930	6.834257	3.583388
H	3.881858	5.371782	4.150611
C	6.059081	2.826299	-3.504717
H	5.481429	1.905855	-3.676340
H	7.116718	2.580740	-3.670417
H	5.748539	3.564397	-4.252196
C	5.776263	-3.195723	3.450621
H	5.280503	-2.225781	3.606942
H	6.848944	-3.041123	3.629903
H	5.393418	-3.897033	4.200184
C	2.736918	-5.939438	-3.445353
H	1.816419	-5.350151	-3.573011
H	2.479680	-6.989543	-3.638549
H	3.457818	-5.609186	-4.201084
C	-6.071041	-2.861306	-3.410830
H	-5.493704	-1.945575	-3.607118
H	-7.129381	-2.618680	-3.576410
H	-5.765172	-3.616929	-4.142558
C	1.196936	2.070228	2.143674
C	-0.246779	2.383051	2.138797
C	-1.172029	1.449019	2.562028
C	1.649561	0.837604	2.572286
C	1.783875	2.695483	0.978837
C	0.733769	3.387279	0.256803
C	-0.513815	3.194342	0.970940
C	-1.711730	3.038198	0.258301
C	-2.378244	1.256285	1.807614
C	-1.664369	-0.848829	2.537279
C	-1.213864	-2.076011	2.098292

C	0.228902	-2.388812	2.103605
C	1.144066	-1.457505	2.546645
C	2.361110	-1.255609	1.812097
C	2.672034	0.161405	1.825388
C	3.271065	0.759876	0.708126
C	2.814834	2.057018	0.274351
C	0.744862	3.415602	-1.139462
C	1.802546	2.737453	-1.867056
C	2.818395	2.071786	-1.172675
C	3.283079	0.775523	-1.633641
C	3.563822	-0.039825	-0.467515
C	3.265555	-1.403830	-0.479232
C	2.662201	-2.028960	0.683179
C	1.713865	-3.014951	0.229495
C	0.508590	-3.182196	0.926964
C	-2.683210	-0.162153	1.794632
C	-1.778573	-2.681591	-1.927409
C	-2.801948	-2.026542	-1.233223
C	-3.261751	-0.722944	-1.679100
C	-2.682837	-0.122947	-2.802433
C	-1.623381	-0.804188	-3.521159
C	0.240001	-2.369234	-3.085782
C	0.518905	-3.180959	-1.919871
C	-0.728320	-3.371681	-1.199454
C	-0.731595	-3.363895	0.197313
C	-1.788954	-2.682636	0.918543
C	-2.813224	-2.033905	0.213857
C	-3.553749	0.074460	-0.502922
C	-3.256660	1.438795	-0.491394
C	-2.655180	2.062625	-1.654264
C	-2.374744	1.297711	-2.790292
C	-1.125056	1.490638	-3.501264
C	-0.661184	0.192343	-3.953659
C	0.706666	-0.104468	-3.948960
C	1.165928	-1.409414	-3.511805
C	1.716040	-3.005247	-1.217294
C	2.676753	-2.010005	-1.658014
C	2.408191	-1.227513	-2.784846
C	2.716593	0.193186	-2.772383
C	1.664477	0.885499	-3.491336
C	1.217992	2.136009	-3.046367
C	-0.203480	2.443917	-3.051642
C	-0.494516	3.236225	-1.875540
C	-1.698855	3.050479	-1.188716
C	-3.272393	-0.743418	0.663092
C	-2.665837	2.046109	0.686662
C	-1.181378	-2.061217	-3.090375
C	0.775171	-0.179834	3.302138
C	1.091995	-0.281042	4.821610
H	1.887836	-1.004522	5.032954
C	-0.803570	0.162170	3.296871
C	-1.144178	0.202914	4.814128
H	-1.029847	1.244012	5.186188
H	-2.169169	-0.127335	5.018503
N	-0.168935	-0.690137	5.418239
C	-0.166690	-0.656843	6.871595
H	-1.147442	-0.972543	7.247950
H	0.593626	-1.348982	7.253791
H	0.051626	0.358444	7.265979
H	1.415381	0.715561	5.192664

Complex B (B97-D2/def2-TZVP)

C	-6.898017	0.963451	-0.106314
C	-6.519280	1.741236	0.984403
C	-5.984977	3.039401	0.837873
C	-5.798319	3.566518	-0.478807
C	-6.399014	2.857361	-1.551974
C	-6.939040	1.603408	-1.374567
C	-5.488220	3.770593	1.973346
C	-4.892098	4.672880	-0.675664
C	-4.295583	5.287512	0.454964
C	-4.701468	4.871550	1.759421
C	-3.159405	6.157345	0.265439
C	-2.586025	6.283796	-1.038903
C	-3.296126	5.773926	-2.181190
C	-4.426085	5.025415	-1.977773
H	-6.493866	1.293979	1.971587
H	-6.396660	3.280012	-2.551696
H	-7.346904	1.077364	-2.233611
H	-4.293390	5.374720	2.630748
H	-4.910092	4.600175	-2.851553
C	-1.258952	6.750319	-1.157835
H	-0.779401	6.667980	-2.126668
C	-2.459981	6.762555	1.343142
H	-2.915988	6.812700	2.326977
C	-0.496805	7.111928	-0.051217
C	-1.173560	7.230123	1.193050
H	-0.649660	7.633641	2.055847
C	0.975678	7.063174	-0.137034
C	1.713909	6.660647	0.972237
C	1.656348	7.126724	-1.382159
C	3.011406	6.115667	0.857718
H	1.230683	6.619234	1.942134
C	2.911919	6.580004	-1.528384
H	1.157410	7.551967	-2.249235
C	3.578090	5.948837	-0.444894
C	3.695267	5.580666	2.005531
H	3.367645	6.593143	-2.513401
C	4.671280	5.023961	-0.625992
C	4.785936	4.774802	1.809809
C	5.233015	4.387358	0.510352
C	5.059306	4.580556	-1.926406
H	5.250141	4.334165	2.686963
C	6.076284	3.231458	0.322483
C	5.776108	3.431007	-2.130854
H	4.682057	5.099232	-2.802421
C	6.221987	2.679059	-0.988853
C	6.639086	2.498167	1.400342
C	6.653545	1.341416	-1.120685
H	6.677129	2.935432	2.393254
C	7.083572	1.205268	1.236262
H	6.584202	0.885236	-2.101475
C	6.975798	0.551063	-0.021180
H	7.461067	0.662014	2.098514
C	-6.963814	-0.505138	0.026932

C	-6.647222	-1.313285	-1.061161
C	-7.065455	-1.138451	1.295455
C	-6.214112	-2.648256	-0.909453
H	-6.583295	-0.872712	-2.049448
C	-6.620827	-2.428746	1.478468
H	-7.438462	-0.580835	2.150484
C	-6.062771	-3.179308	0.410133
C	-5.771254	-3.417803	-2.040883
H	-6.654495	-2.849816	2.478507
C	-5.217653	-4.331289	0.613302
C	-5.053135	-4.563633	-1.820669
C	-4.660650	-4.986436	-0.514780
C	-4.763001	-4.695268	1.916891
H	-4.677520	-5.095178	-2.689648
C	-3.566849	-5.908555	-0.323178
C	-3.670086	-5.496074	2.120803
H	-5.222150	-4.238998	2.788671
C	-2.992857	-6.052000	0.979024
C	-2.907560	-6.560370	-1.398603
C	-1.695204	-6.596082	1.096171
H	-3.369305	-6.591504	-2.380436
C	-1.651595	-7.105506	-1.249482
H	-1.206449	-6.537182	2.062355
C	-0.963653	-7.019445	-0.009728
H	-1.157947	-7.547114	-2.111399
C	6.909718	-0.919589	-0.129981
C	6.540974	-1.680583	0.975944
C	6.939674	-1.579450	-1.388414
C	6.005011	-2.980693	0.854226
H	6.524441	-1.218375	1.956439
C	6.398156	-2.835845	-1.541578
H	7.340142	-1.067216	-2.259172
C	5.806980	-3.528350	-0.452340
C	5.517078	-3.693530	2.005138
H	6.387283	-3.273788	-2.534605
C	4.900005	-4.638135	-0.624391
C	4.726868	-4.796428	1.814947
C	4.310936	-5.233314	0.520431
C	4.426428	-5.013890	-1.917347
H	4.325229	-5.285185	2.697409
C	3.173250	-6.105697	0.353128
C	3.295632	-5.766171	-2.100769
H	4.905407	-4.604717	-2.801494
C	2.592366	-6.255611	-0.945448
C	2.480148	-6.691743	1.445420
C	1.264984	-6.724962	-1.048545
H	2.941563	-6.724037	2.427459
C	1.193162	-7.162563	1.311020
H	0.779941	-6.660525	-2.016029
C	0.509300	-7.067062	0.068644
H	0.674338	-7.550627	2.183918
C	-3.132717	-5.712733	3.512839
H	-2.158976	-5.216556	3.639101
H	-2.980618	-6.779884	3.724202
H	-3.820458	-5.301872	4.259912
C	-5.752753	3.284130	3.375584
H	-5.243444	2.326709	3.560805
H	-6.824743	3.118127	3.548371
H	-5.387575	4.009346	4.110852
C	-2.748934	5.962135	-3.572839
H	-1.811979	5.399820	-3.700643
H	-2.523862	7.017122	-3.779328
H	-3.464013	5.602748	-4.320717
C	3.167197	5.824614	3.396614
H	2.194368	5.331170	3.539663
H	3.016365	6.895772	3.587630
H	3.860160	5.428882	4.147023
C	5.997629	2.910035	-3.527917
H	5.410359	1.995762	-3.699362
H	7.051680	2.655136	-3.702084
H	5.689197	3.654076	-4.270346
C	5.794149	-3.185788	3.397418
H	5.287239	-2.225154	3.572538
H	6.867729	-3.018143	3.558297
H	5.434846	-3.899335	4.146862
C	2.740097	-5.978734	-3.485575
H	1.802622	-5.418207	-3.617400
H	2.513438	-7.037054	-3.672316
H	3.450743	-5.632673	-4.243901
C	-5.996787	-2.918529	-3.445145
H	-5.412618	-2.005167	-3.631518
H	-7.051927	-2.669387	-3.621169
H	-5.687610	-3.672785	-4.176878
C	1.339086	2.046331	2.184274
C	-0.052001	2.463151	2.171045
C	-1.050785	1.583225	2.602978
C	1.680977	0.764741	2.630785
C	1.986903	2.604130	1.011412
C	0.996763	3.367495	0.273688
C	-0.264150	3.279022	0.989255
C	-1.467176	3.180058	0.283470
C	-2.301009	1.484581	1.871337
C	-1.726406	-0.667196	2.618708
C	-1.378028	-1.956421	2.200090
C	0.012980	-2.373338	2.214760
C	1.005373	-1.485673	2.645430
C	2.266169	-1.399962	1.930495
C	2.684217	-0.008729	1.921578
C	3.307044	0.527823	0.790308
C	2.951555	1.857142	0.327431
C	1.006655	3.353986	-1.123472
C	2.005792	2.576176	-1.832660
C	2.959004	1.842034	-1.121666
C	3.322442	0.506027	-1.555706
C	3.535080	-0.308378	-0.373523
C	3.131903	-1.646415	-0.364540
C	2.485197	-2.203604	0.808406
C	1.455871	-3.124056	0.361301
C	0.242451	-3.210074	1.050873
C	-2.719143	0.093354	1.881288
C	-1.985645	-2.558307	-1.816035
C	-2.949176	-1.811733	-1.132230
C	-3.305987	-0.483728	-1.595335
C	-2.684048	0.052314	-2.727413
C	-1.681509	-0.720864	-3.437514
C	0.051298	-2.418294	-2.976620

C	0.263381	-3.233195	-1.794333
C	-0.997100	-3.323134	-1.078493
C	-1.007746	-3.311410	0.318657
C	-2.008496	-2.535147	1.028070
C	-2.963132	-1.800895	0.316968
C	-3.535800	0.351652	-0.431016
C	-3.132456	1.689578	-0.440167
C	-2.484392	2.245388	-1.613343
C	-2.266578	1.443437	-2.736870
C	-1.006350	1.528828	-3.452705
C	-0.645047	0.191585	-3.885575
C	0.695340	-0.210383	-3.872207
C	1.050077	-1.539631	-3.410080
C	1.465668	-3.134360	-1.088428
C	2.500947	-2.223156	-1.536820
C	2.299647	-1.441361	-2.677538
C	2.717256	-0.050305	-2.687038
C	1.725207	0.709993	-3.425342
C	1.376336	1.998455	-3.005823
C	-0.013854	2.415209	-3.020416
C	-0.243110	3.251093	-1.856015
C	-1.455538	3.164419	-1.166121
C	-3.325008	-0.463520	0.750675
C	-2.502960	2.267747	0.731796
C	-1.338982	-2.001604	-2.989946
C	0.644094	-0.147977	3.077750
C	-0.696095	0.253503	3.064462
